# Decoy Oligonucleotide Rescues IGF1R Expression from MicroRNA-223 Suppression

**DOI:** 10.1371/journal.pone.0082167

**Published:** 2013-12-04

**Authors:** Li Hui Wu, Qian Qian Cai, Yi Wei Dong, Rong Wang, Bao Mei He, Bing Qi, Chang Jun Xu, Xing Zhong Wu

**Affiliations:** 1 Department of Children’s Health Care, Yu Ying Children’s Hospital, Wenzhou Medical College, Wenzhou, China; 2 Department of Biochemistry and Molecular Biology, Shanghai Medical College, Fudan University, Key Laboratory of Glycoconjugate Research, Ministry of Public Health, Shanghai, China; 3 Department of Biochemistry and Molecular Biology, Guiyang college of traditional Chinese medicine, Guizhou province, China; University of Toronto, Canada

## Abstract

A mature miRNA generally suppresses hundreds of mRNA targets. To evaluate the selective effect of synthetic oligonucleotide decoys on hsa-miR-223 activity, reporters containing 3’ untranslated regions (UTR) of IGF1R, FOXO1, POLR3G, FOXO3, CDC27, FBXW7 and PAXIP1 mRNAs were constructed for the luciferase assay. The oligonucleotide decoys were designed and synthesized according to mature miR-223 sequence and its target mRNA sequence. Quantitative RT-PCR & western analysis were used to measure miR-223-targeted mRNA expression, Interestingly, apart from the antisense oligonucleotide, decoy nucleotides which were complementary to the 5’, central or 3’ region of mature miR-223 suppressed miR-223 targeting the 3’UTR of IGF1R, FOXO1, FOXO3, CDC27, POLR3G, and FBXW7 mRNAs and rescued the expression of these genes to varying degrees from miR-223 suppression at both mRNA and protein levels. All decoys had no effect on PAXIP1 which was not targeted by miR-223. The decoy 1 that was based on the sequence of IGF1R 3’UTR rescued the expression of IGF1R more significantly than other decoy nucleotides except the antisense decoy 4. Decoy 1 also rescued the expression of FOXO3 and POLR3G of which their 3’UTRs have similar binding sites for miR-223 with IGF1R 3’UTR. However decoy 1 failed to recover Sp1, CDC27 and FBXW7 expression. These data support that the sequence-specific decoy oligonucleotides might represent exogenous competing RNA which selectively inhibits microRNA targeting.

## Introduction

MicroRNA (miRNA) is a single-stranded, non-coding RNA molecule of 22–25 nucleotides, which belongs a family of regulatory molecules involving cell development, differentiation, apoptosis, proliferation and even in tumorigenesis [1,2]. In RNA transcript, the active region of the miRNA is contained within an about 70-nucleotide hairpin structure (pre-miRNA), which is cleaved by the endonuclease Dicer to yield a ^~^21-nucleotide miRNA duplex [1]. One stand is then degraded; the other is referred to as the ‘mature’ miRNA. Although either strand may act as functional miRNA, only one strand is usually incorporated into the RISC (RNA-induced silencing complex) where the miRNA and its mRNA target interact. In cases of complete or partially complementary pairing between miRNA and its target sequences, the complex RISC induces transcript cleavage [2]. As the base pairing between miRNAs and mRNA 3’ untranslated region (UTR) in a sequence-specific manner [3], it is conceivable that miRNAs can interfere with the activity of RNA-binding proteins. This results in inhibition of translation initiation [4] and/or rapid deadenylation [5] of the target transcript. Particularly oligonucleotides with the sequence complementary to the mature miRNA also induce miRNA transcript cleavage and expression silence. Haraguchi [6] optimized the entire secondary structure of some anti-miRNA oligonucleotides and the sequence of miRNA-binding site, which lead to longer and persistent inhibition. However, miRNA can target hundreds of mRNA molecules and interfere with their stability and protein translation through sequence matching [7]. Therefore, loss of function in one important miRNA will lead to alteration of hundreds of target mRNAs among which their diverse cellular functions impair the regulation balance. Recent reports [8,9] show that the 3’UTR of an mRNA can modulate the activities of miRNA in the cytoplasm due to base paring. Oligonucleotides with a similar sequence to the target 3’UTR as a decoy oligonucleotide are supposed to bind to the corresponding mature miRNA competitively. Decoy RNA molecules were thus predicted to be able to target mature miRNA as exogenous competing RNA (cRNA) and protect the corresponding mRNAs selectively. In this study, we carried out a systematic study to evaluate the selective targeting effect of synthetic decoys on hsa-miR-223 activity. Panels of decoys containing base-pairing sites with both the sequence of mature-miR-223 and the target mRNA 3’UTRs were tested against miR-223 activity. 

## Materials and method

### Cell culture and transfection

HeLa and HEK-293T cells were obtained from ATCC. Hepatocellular carcinoma cells (SMMC-7721 and LM3) and liver adenocarcinoma (SK-hep-1) cells were obtained from Biochemistry and Cell biology Institute of Shanghai, Chinese Academy of Science. These cells were cultured in the medium of DMEM supplemented with 10% newborn calf serum (PAA, Australia) or fetal bovine serum (Gibco, Carlsbad, CA, USA) and plated in serum-free media without antibiotics in 96-well plates(1×10^4^ cells/well) 24h before transfection. The plasmids pLL 3.7-pre-miR-223 and psiCHECK-2 containing target mRNA 3’UTR sequence or psiCHECK-2 control vector were co-transfected with Lipofectamine 2000 (Invitrogen, Carlsbad, CA, USA) at the ratio of 1:3 in weight [10]. The mixtures were diluted to wells that already contained the decoy oligonucleotides. 1 OD decoy oligonucleotides were diluted in 250 μl Opti-Mem (20 μM) provided by the company. All the cells were grown at 37°C and the medium was refreshed after 24h. The cells were harvested 24 - 48h after transfection for luciferase assay, qPCR, Western blot and other assays. All transfection experiments were carried out in triplicate.

### The sequence of decoys

**Figure 1:**
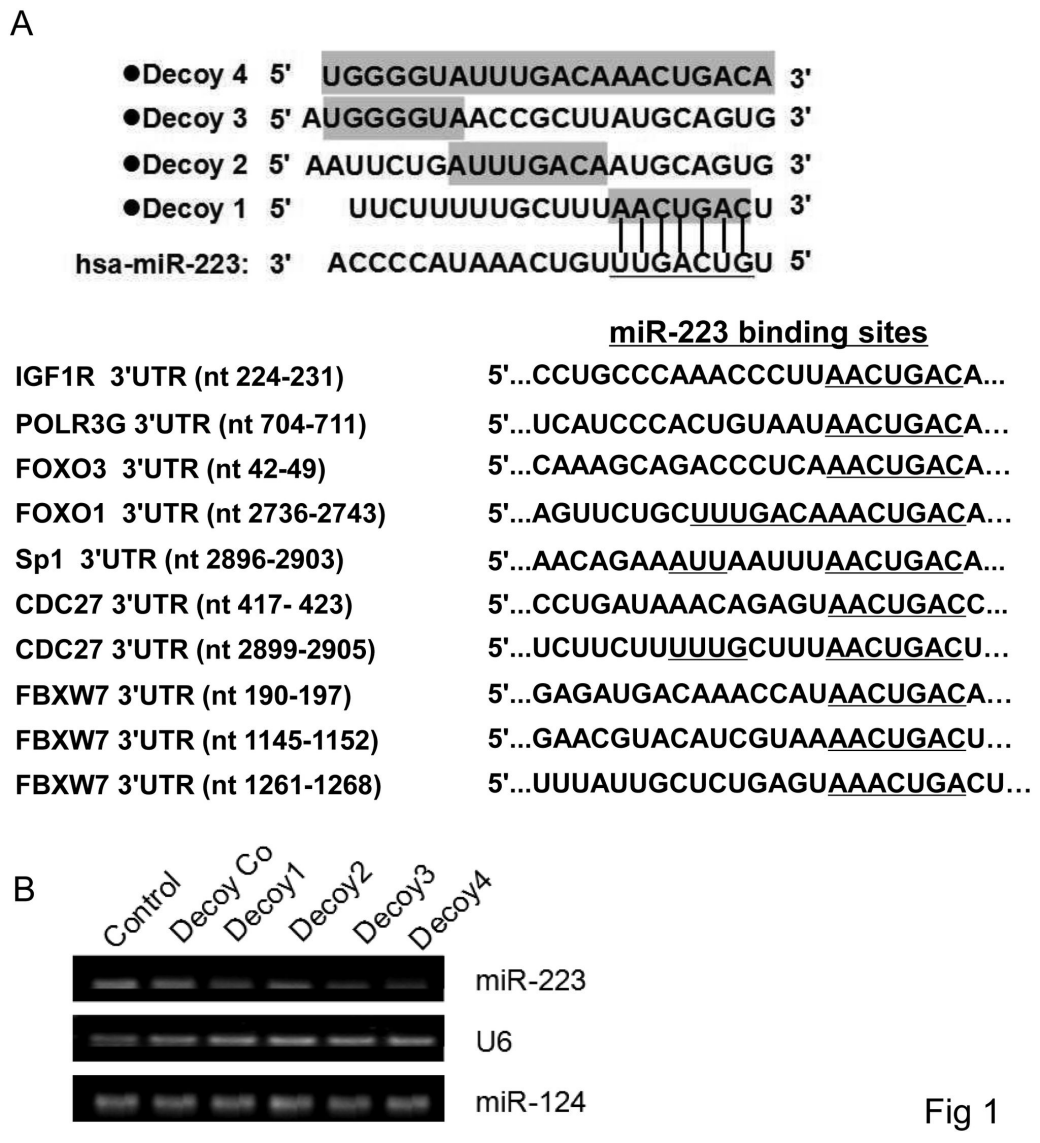
Matching sequence of miR-223 with the 3’ UTR of predicted targets and decoy nucleotides. **A**. Four decoy nucleotides were designed and the decoy sequence complementary to miR-223 is shown in shade (top). There is one putative miR-223 binding site in the 3’ UTR of IGF1R mRNA, which is located at the region from 224 to 231 nt from the stop codon, while FOXO1 at 2736 to 2743 nt, and POL3G at 704 - 711 nt, respectively. CDC27 and FBXW7 mRNAs have two and three putative binding sites of miR-223 (bottom). **B**. Expression measurement of miR-223 and miR-124 by RT-PCR in SMMC-7721 cells transfected with decoys as indicated. Data are representative of 3 independent experiments. Co, control.

Based on the sequence of mature hsa-miR-223 (from Sanger Institute miRBase) and of the target mRNAs, decoy oligonucleotides which were used to target mature miR-223 were designed and the sequence of the decoy oligonucleotides was listed in the Figure 1. The decoy and their scramble control oligonucleotides were then synthesized and provided by Gene Pharma Co.,Ltd (Shanghai, China) .

### Lentivirus vector containing pre-miR-223

The fragment encoding the hsa-pre-miR-223 sequence plus 150bp at both 5’- and 3’-flanking regions was cloned from genomic DNA from NB4 cells and inserted into the *Hpa I* and *Xho I* sites of lentivirus pLL3.7 vector which was used to express miR-223[10].

### Luciferase assay

Renilla luciferase assay was used to assess the effect of miR-223 on the 3’UTR fragment transcript stability and translation efficiency [11], and the firefly luciferase served as control. 48h after the transfection, the cells in the 24-well plate were washed with PBS twice and lyzed in 100 μl/well Passive Lysis Buffer (Promega, Madison, WI, USA). Firefly and Renilla luciferase activities in 15 μl lysate were measured using the Dual-Glo Luciferase Assay System (Promega, Madison, WI, USA) with an illuminometer (Lumat LB 9507, Berthold, Germany) according to manufacturer’s instructions. All the data were obtained by averaging the results from three independent repeats.

### RNA isolation and quantitative polymerase chain reaction

Total RNA was isolated from cells using Trizol regent (Invitrogen, CA, USA), Reverse transcription was performed based on our previous report [10]. 200 ng of total RNA from each sample was used for primer-specific reverse transcription (RT). 2 μl of the reverse transcription product was used for subsequent quantitative polymerase chain reaction (qPCR), Detection of mature miR-223 was performed with stem-loop PCR using SYBR GreenMaster Mix (DBI Bioscience, Shanghai, China) according to the protocol based on our previous report [12]. The specific primers were listed at the table 1. The qPCR was then performed with the iQ5^TM^ quantitative PCR detection system (Bio-Rad, Hercules, CA, USA), and data were averaged and normalized to RNU6 levels. The gene expression levels rather than miRNA were normalized as fold change relative to the housekeeping gene, GAPDH. All assays were run in triplicate and data were collected from 3 independent experiments.

**Table 1:** 

Specific primers for reverse transcription	
miR-223	5’-GTCGTATCCAGTGCAGGGTCCGAGGTATTCGCACTGGATACGACTGGGGT-3’
miR-124	5’-GTCGTATCCAGTGCAGGGTCCGAGGTATTCGCACTGGATACGACGGCATTC-3’
RNU6	5’-AAAATATGGAACGCT-3’
Primers for qPCR	
miR-223	5’-GTGCAGGGTCCGAGGT-3’ 5’-CGGGCTGTCAGTTTGTCA-3’
miR-124	5’-GTGCAGGGTCCGAGGT-3’ 5’ -ACAATTAAGGCACGCGGTG-3’
RNU6	5’-CTCGCTTCGGCAGCACA-3’ 5’-AACGCTTCACGAATTTGCGT-3’
Primers for 3’UTR cloning	
IGF1R	5’-CCCCCTCGAGGATCCTGAATCTGTGCAAAC-3’ 5’-AAAAGCGGCCGCCTTCCCAGCGAAATCATC-3’
Sp1	5’- TCACAAGCCAGTTCCAGCTCC-3’ 5’- GGGTGCACCTGGATTCCTGAA -3’
CDC27	5’-CCCCCTCGAGTCTGGAAATCAGACTTTTAC-3’ 5’-AAAA GCGGCCGC CATATCCTTTAATGAATTGC-3’
Foxo1	5’-AAAA CTCGAGGGGTTAGTGAGCAGGTTAC-3’ 5’-AAAA GCGGCCGC GTGGCTGACAAGACTTAACT-3’
Foxo3	5’-AAAA CTCGAGAGGATCACTGAGGAAGGGG-3’ 5’-AAAA GCGGCCGC AGAAAGCACCTATACAGCACC-3’
FBXW7	5’-CCCC CTCGAGAGAGCAGAAAAGATGAATT-3’ 5’-CCCC GCGGCCGC TAACATGAAAAAACACATTT-3’
POLE3G	5’-CCCC CTCGAGGCATGAAATTTTTCAAAAAAT-3’ 5’-CCCC GCGGCCGC TTGTTTGTGGTGATTGTTTAT-3’

### Western blotting

Fifty micrograms of total proteins from each sample was loaded onto 10% sodium dodecyl sulfate-polyacrylamide gel electrophoresis, then transferred onto polyvinylidene difluoride (PVDF) membranes (Millipore, Billerico, Massochusatts, USA). After blocking with 5% bovine milk protein, the blots were incubated with antibodies against IGF1R, caspase-3 (Bioworld, St. Louis Park, MN, USA), Sp1 (Santa Cruz, CA, USA) and glyceraldehyde-3-phosphate dehydrogenase (GAPDH) at 4°C overnight. After incubation with horseradish peroxidase-conjugated secondary antibody at room temperature for 2h, protein bands were visualized by enhanced chemiluminescence detection kit (Pierce, Thermo, Rockford, IL, USA). The image was captured by the auto-image system with an EM CCD camera (Tenon, Shanghai, China) and the density of the bands was analyzed and summarized.

### Cell Growth and metabolic activity Assays

MTT (3-(4, 5-dimethylthiazol-2-yl)-2, 5-diphenyltetrazolium bromide) [13] and CCK-8 (Cell Counting Kit-8 assay, Shanghai R&S Biotechnology, China) assays [14] were used to measure viable cells. Hepatoma cells (LM3), liver adenocarcinoma (SK-hep-1) cells and NB4 promyelocytic leukemia cells (5 × 10^3^ cells/well) were cultured in 96-well plates for the measurement of cell growth or metabolic activity of viable cells. Decoy oligonucleotides (50 nM) were transiently transfected into cells with Lipofectamine 2000. 48h after incubation, the medium was then removed and a mixture comprising 10 μl of the 10 mM MTT or CCK-8 stock solution and 90 μl of fresh medium was added to each well of the 96-well plate. The plates were then incubated for additional 4h at 37°C, and DMSO was added to the wells and mixed by a shaker to dissolve the dark blue formazan product from MTT. The optical density at 570 nm of each well was determined using a microplate reader over 620 nm. For CCK-8 assay, the absorbance at 450 nm was determined after 24h incubation according to manufacturer’s instruction. The optical density value representing cell viability and number in each group was calculated and summarized from 3 independent experiments.

### Cell Adhesion Assay

The adhesion assay was performed as previously reported[13]. Briefly, 96-well plates were coated at 100 μl/well with collagen type I (40 µg/ml) (Roche, Indianapolis, IN, USA), fibronectin (10 µg/ml) (Calbiochem, San Diego, CA) and heparin (1mg/ml) at 4°C overnight. The wells were coated with 300 ug/ml poly-lysine or 1%BSA as positive or negative control. After blocking with 1% BSA for 30min at 37 °C and washing twice with PBS, 100 µl of the cell suspension (4×10^4^/ml) was added to each well. After incubation at 37 °C with 5% CO_2_ for 2h, the medium was removed and all the wells were washed with PBS twice, stained with MTT. The absorbance value was measured at 570 nm over 630 nm to represent adhesion cell number. The adhesion rate was calculated by the formula (A570 value in test group - A570 in negative group) / (A570 in positive group - A570 in negative group) × 100%. 

### Acidic Phosphatase Assay

Cells were seeded and cultured in 96-well plates with an initial density of 1000 cells per well. 24h after transfection the plates were washed with PBS, and each well of the plates was added with 100 μl reaction solution (0.1 mol/l NaAc pH 5.0, 0.1% Triton X-100, 50mM Tris, 5 mmol/l p-nitrophenyl phosphate) .After incubation at 37°C for 2 h, the reaction was stopped by adding 10μl 1N NaOH per well and the absorbance at 450 over 630 nm was read. The activity of the acidic phosphatase represented the number of survival cells tested as previous reports [14] .

### Colony formation assay

The method was according to our previous report [13]. Briefly, cells were suspended into a single cell status. 1×10^4^ cells from each group were seeded in the culture dish (60 mm diameter) with 10% FBS and incubated for 10 days. The colonies were then fixed with 4% paraformaldehyde and stained with 0.5% crystal violet for 10 min. The colony consisting of more than 40 cells was defined as one colony. The number of colonies in 10 random view fields of each dish was counted under a microscope and the average representing the 95% confident region was achieved. 

### Hoechst staining

SMMC-7721 cells co-transfected with miR-223 and decoy nucleotides were plated on cleaned cover-glass in 6-well plates. After 48h, the medium was replaced by serum-free medium and continue to incubate 12h to induce cell apoptosis. Then the cells on the slides was stained by the Hoechst staining kit (Beyotime, Shanghai, China) and examined under a fluorescent microscope.

### Data analysis

Quantitative data were expressed as the means with SD of at least three independent experiments. The levels of miRNAs or mRNA expression were represented as fold changes after normalizing over the internal controls. The one-way ANOVA was used to evaluate the difference in experimental data among multiple groups with SPSS software version 11.0 and Student’s t test was for the analysis of statistical difference between 2 groups. *P* values less than 0.05 were considered significance.

## Results

### Designing of miR-223 targeting decoys

Through analysis using online algorithms, miRanda (http://microrna.sanger.ac.uk/sequences/index.shtml), TargetScan (http://www.targetscan.org), Pictar (http://pictar.bio.nyu.edu) and microRNA.org. (http://www.microrna.org/), several mRNAs with conserved target sites were predicted to match miR-223 seed sequence (Figure 1A). The predicted targets of miR-223 included insulin-like growth factor 1 receptor (IGF1R), F-box and WD repeat domain containing 7 (FBXW7), forkhead box O1 (FOXO1), forkhead box O3 (FOXO3), DNA directed RNA polymerase III polypeptide G (POLR3G), cell division cycle 27 (CDC27), and specificity protein 1 (Sp1), among which IGF1R, FOXO1 and FBXW7 were previously reported by us[10,12] and others[15]. The 3’UTR (untranslated region) of all these 7 candidate mRNAs was then amplified by PCR and inserted into *Xho 1* and *Not 1* sites immediately downstream of the Renilla luciferase gene in the dual-luciferase plasmid psiCHECK-2 vector that contains both synthetic firefly and Renilla luciferase genes in a single vector. The decoy nucleotides have the sequence not only complementary to miR-223 seed sequence, but similar to the sequences of IGF1R and FOXO1 3’ UTR where the sequence was putatively targeted by miR-223. The decoy containing the sequence of 3’UTR of IGF1R mRNA and complementary to the seed of miR-223 was assigned as decoy 1 or IGF1R decoy. The decoy containing the sequence complementary to the central part of mature miR-223 and similar to the 3’UTR of FOXO1 mRNA was assigned as decoy 2 or FOXO1 decoy. The decoy containing the sequence complementary to the 3 prime region of mature miR-223 was assigned as decoy 3, and the decoy containing the sequence perfectly complementary to mature miR-223 was assigned as decoy 4.

### Decoys Inhibit miR-223 Targeting

**Figure 2:**
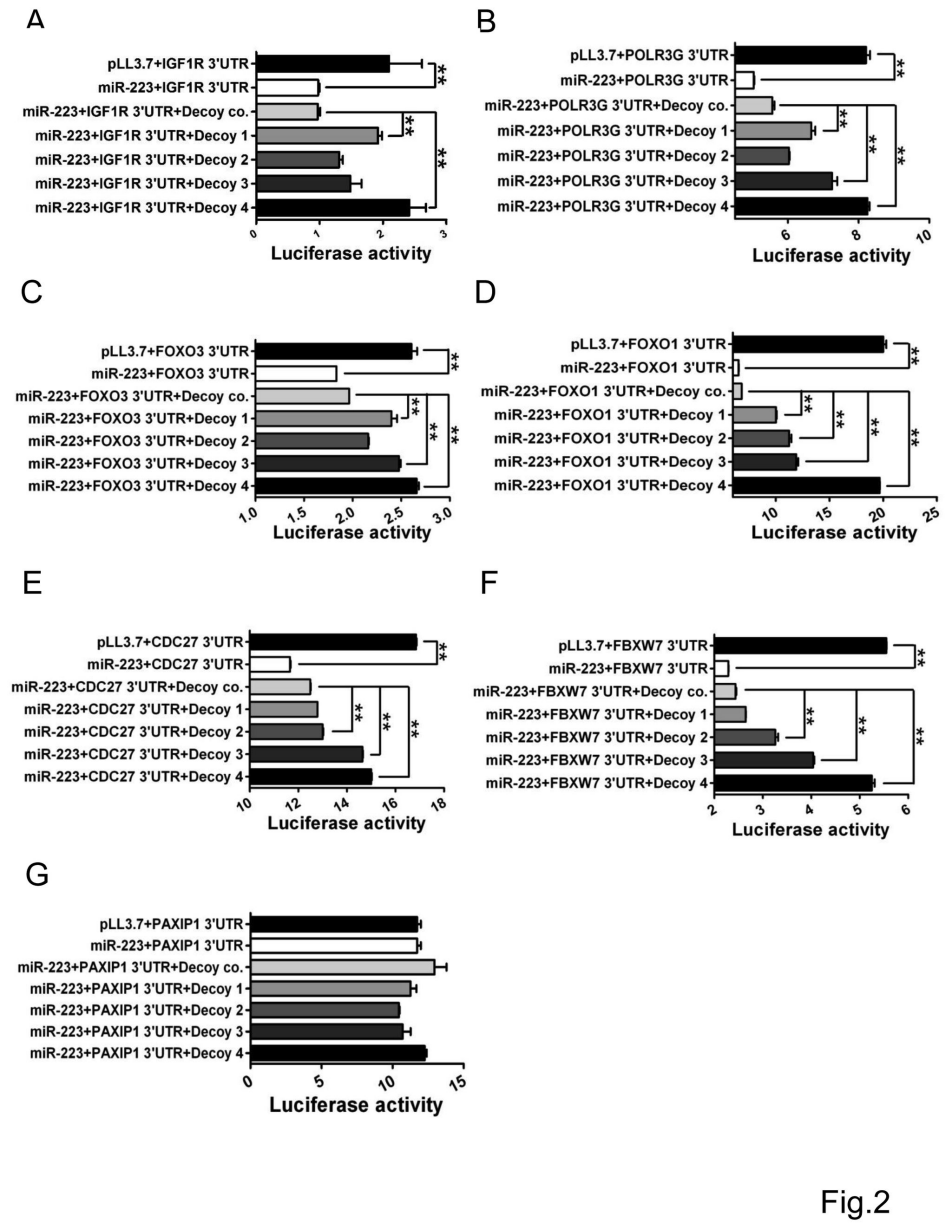
Observation of decoy nucleotide influence on miR-223 targeting the 3’UTR by dual luciferase assay. Reporter luciferase activities were measured in the HeLa cells co-transfected with the decoy nucleotides (25 μM), pLL3.7-miR-223, and 3’UTR constructs {psiCHEK-2-3’UTR of IGF1R (A), POLE3G (B), FOXO3 (C), FOXO1 (D), CDC27 (E), FBXW7 (F), PAXIP1 (G)} or the control nucleotide + pLL3.7-miR-223 + psiCHEK-2-3’UTR constructs. Renilla luciferase activities are normalized over firefly luciferase activities and are summarized as mean with the standard deviation. Data are representative from 3 independent experiments. Co. control. **, *P*<0.01.

In order to validate the effects of the decoys designed in this study, we detected the expression of endogenous miR-223 and miR-124 by RT-PCR in SMMC-7721 cells. After transfection of the decoys, the level of miR-223 expression was down regulated compared to controls, but miR-124 did not (Figure 1B). We then tested the influence of the decoy nucleotides on miR-223 targeting the 3’UTR based on the pairing between miR-223 and its target sequence. The activities of the decoys were monitored by measuring the relative expression levels of reporter luciferase (Renilla) to their control luciferase (firefly) activities. In each case, as compared to the control cells, transfection of miR-223 indeed significantly inhibited the luciferase activities in all groups of the reporters containing 3’UTR of IGF1R, FOXO3, POLR3G, FOXO1, CDC27, and FBXW7 (Figure 2). While, co-transfection of the decoy oligonucleotide with miR-223 construct led to an increase in the luciferase activity in all assays compared to the group with miR-223 alone, suggesting the effective inhibition of miR-223 activity and expression rescuing of the targets. However, the rescuing effect was not always the same among all these 4 decoy nucleotides. The decoy 1, interestingly, inhibited miR-223 targeting IGF1R 3’UTR more efficiently than the decoy 2 in either HeLa cells (Figure 2A) or HEK-203T cells (data not shown), The inhibitory effect of decoy 1 on miR-223 targeting POLR3G and FOXO3 3’UTR was also similar to IGF1R (Figure 2B,C) as they had the same seed complements. The luciferase activity significantly increased after co-transfection of decoy 1 with miR-223 although the luciferase activity was not recovered to the level of control vector group. There was no significant difference of luciferase activity between the groups of decoy 2 + miR-223 and miR-223 in IGF1R, POLR3G and FOXO3 3’UTR reporter assay. However, decoy 2 had stronger inhibitory effect on miR-223 targeting FOXO1 3’UTR (Figure 2D). Similarly, the decoy 2 recovered CDC27 mRNA 3’UTR from the inhibition by miR-223 more significantly than the decoy 1(Figure 2E) as CDC27 shared two similar additional complementary bases with FOXO1 besides the seed complementary sequence. The rescue effect on FBXW7 3’UTR from the inhibition by miR-223 was quite different from the others. Co-transfection with decoy 1 did not significantly elevate the luciferase activity inhibited by miR-223 (Figure 2F), and only showed slight rescuing effect on FBXW7 3’UTR, but decoys 2 and 3 offered significant rescue effect on FBXW7 3’UTR (Figure 2F). Among all the 3’UTRs that we tested in this study, the relative luciferase activities are highest in the group of decoy 4, suggesting that the nucleotide completely complementary to mature miR-223 was the most potent and strongest rescuer of all the 3’UTRs. Furthermore, decoy 3 containing sequence partially complementary to 3’ region of mature miR-223 had also a robust role in recovering the targets from miR-223 repression (Figure 2). All the decoys had no any effect on the activities of the reporter carrying 3’UTR of PAXIP1 mRNA which was not targeted by miR-223 (Figure 2G), suggesting the rescue effect of the decoy was specific.

### Decoys strengthen miR-223-targeted mRNA expression

**Figure 3:**
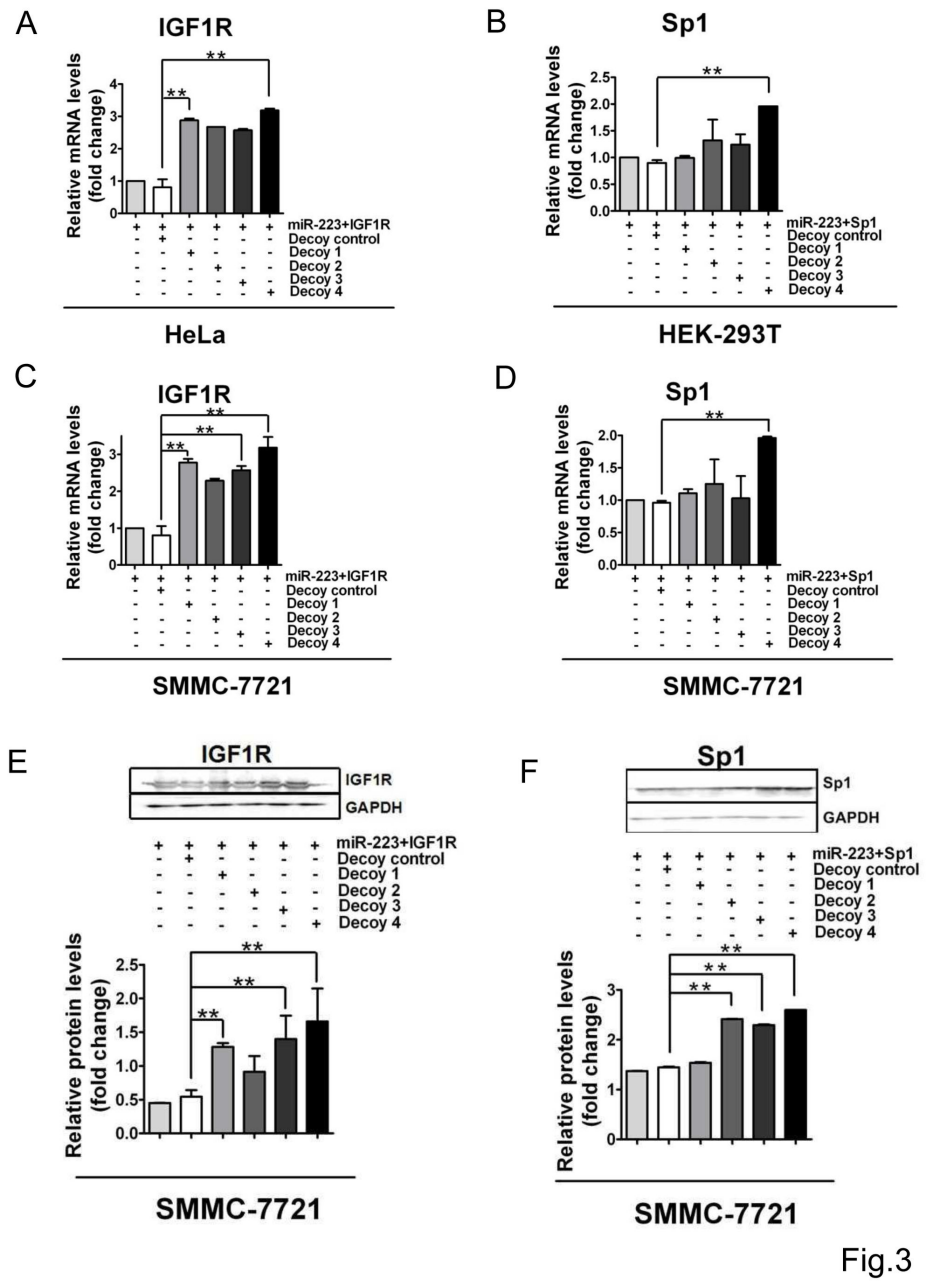
IGF1R expression rescue from miR-223 suppression. **A**&**B**. The mRNA expression of IGF1R (A) and Sp1 (B) was monitored by qRT-PCR examination in HeLa or HEK-293T cells transfected with pLL3.7-miR-223, psiCHEK-2-IGF1R3’UTR and decoy nucleotides. The expression levels were normalized to GAPDH which served as the loading control. **C**&**D**. The mRNA expression of IGF1R (C) and Sp1 (D) was measured in SMMC-7721 cells transfected with pLL3.7-miR-223, psiCHEK-2-IGF1R3’UTR and decoy nucleotides. **E**&**F**. The protein levels of IGF1R (C) and Sp1 (D) in SMMC-7721 cells co-transfected with miR-223 and decoy nucleotides (upper), and quantified by densitometry (lower). GAPDH served as an interior loading control. The data are representative from 3 independent experiments. **, *P*<0.01 .

In order to confirm the inhibitory effect of decoys on the miR-223 targeting, we further performed qRT-PCR and western analysis to monitor the mRNA and protein expression of the target molecules. We first observed the influence of the decoys on the mRNA expression of IGF1R and Sp1 in two cell lines, HeLa and HEK-293T, respectively. Ectopic expression of miR-223 resulted in repression of either IGF1R or Sp1 mRNA expression, but decoys significantly increased IGF1R mRNA expression (Figure 3A), especially decoy 1, although, decoy 4 complimentary to the whole miR-223 sequence rescued IGF1R mRNA expression the best. The expression of Sp1 mRNA was strongly rescued by decoy 4, but not significantly by other decoys (Figure 3B). Similar results were seen in SMMC-7721 cells (Figure 3C, D).,

Meanwhile, the protein level of IGF1R was measured by western analysis and the result showed that IGF1R was significantly rescued by decoy 1 as well as decoy 3 or 4 (Figure 3E), but decoy 2 did not. However, decoy 1 failed to elevate the expression level of Sp1 protein in SMMC-7721 cells significantly when miR-223 was over-expressed (Figure 3F), while decoys 4, 3, and 2 rescued Sp1 protein level in miR-223-overexpressing cells. 

### Regulation of miR-223 by decoys involves cell growth

**Figure 4:**
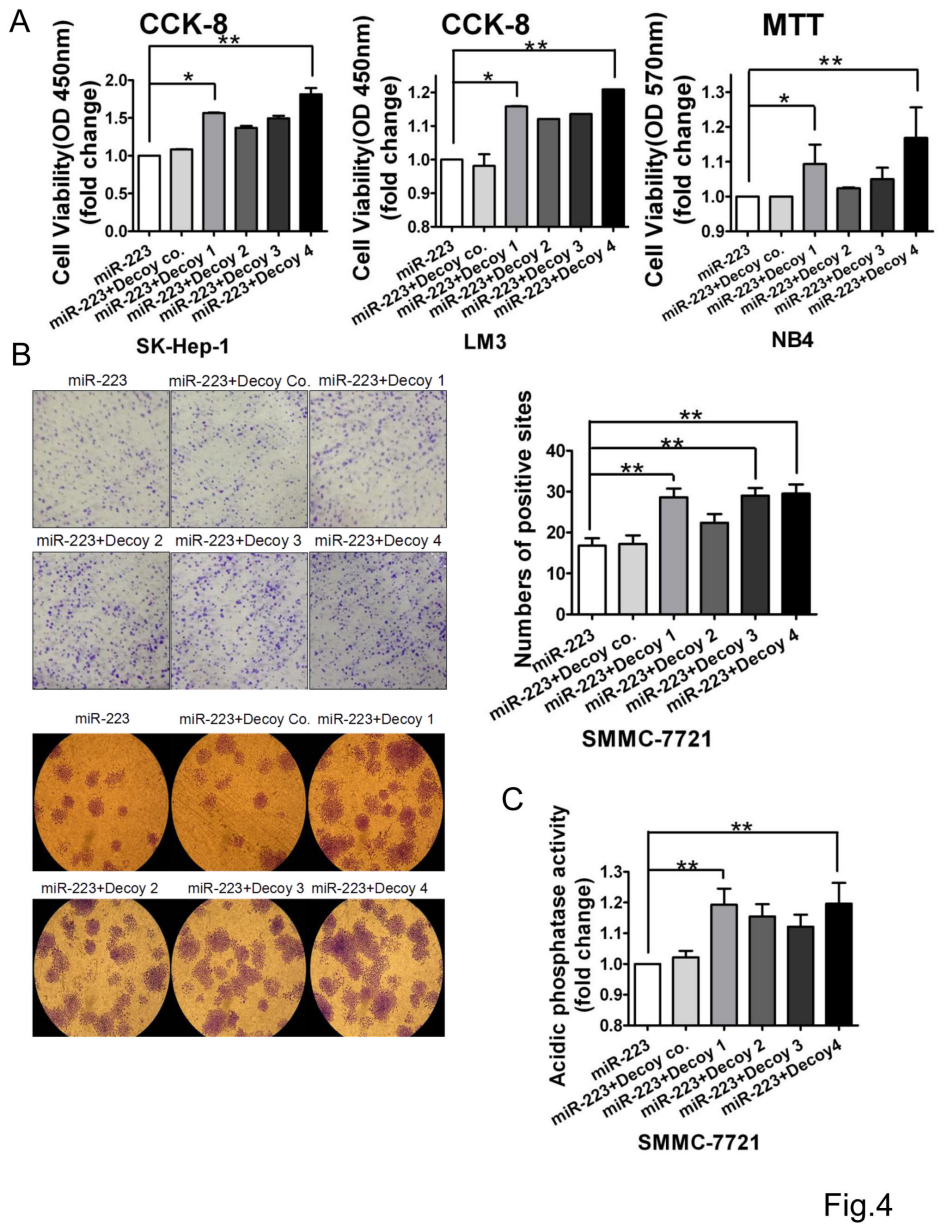
miR-223-mediated cell growth suppression was reversed by decoy nucleotides. **A**. The cell growth rate of SK-hep-1 (left) and LM3 (middle) cells was evaluated by CCK-8 assay. The optical density value at 450 nm represents the viable cell number was summarized from 3 independent experiments with standard error. The metabolic activity of NB4 cells (right) were measured by MTT assay. The optical density value at 570 nm represents the viable cell number was summarized from 3 independent experiments with standard error. **B**. Colony-forming analysis of SMMC-7721 cells transfected with miR-223 and decoys. Representative images of the colonies on culture dishes (upper, left). Representative images of micrograph (lower, 4X) of the colonies. The colonies are quantified by counting (right). The number of colonies in 10 random view fields was counted under a microscope and the average representing 95% confidence was achieved. **C**. The activity of acidic phosphatase measured in SMMC-7721 cells co-transfected with miR-223 and decoys, and summarized as fold changes. The data are representative of 3 independent experiments. *, *P*<0.05, **, *P*<0.01, co, control.

Regulation of miR-223 in hepatocellular carcinoma cells involves cell proliferation in our previous observation[10]. Hence, we further investigated the effect of these decoys on the cell growth though CCK-8, MTT, acidic phosphatase and colony formation assays in hepatoma cells (LM3, SK-Hep-1, SMMC-7721). The results of the CCK-8 assays showed that decoy 1 and decoy 4 increased viable cell numbers and rescued cell growth which was inhibited by miR-223 in either SK-hep-1 or LM3 cells (Figure 4A). Although decoy 2 and decoy 3 rescued the growth in these two cells, there was no significant difference between test group and control (Figure 4A). In promyelocytic leukemia NB4 cells which highly express miR-223, decoys 1 and 4 significantly rescued the cell proliferation respectively (Figure 4A), but decoy 3 or 2 not. Results of the colony formation and acidic phosphatase assays also showed that decoys 4 and 1 restored the cell growth from miR-223-mediated suppression (Figure 4B,C), while decoys 2 and 3 only slightly. These data were consistent with the regulatory role in rescuing IGF1R expression presented above. 

**Figure 5:**
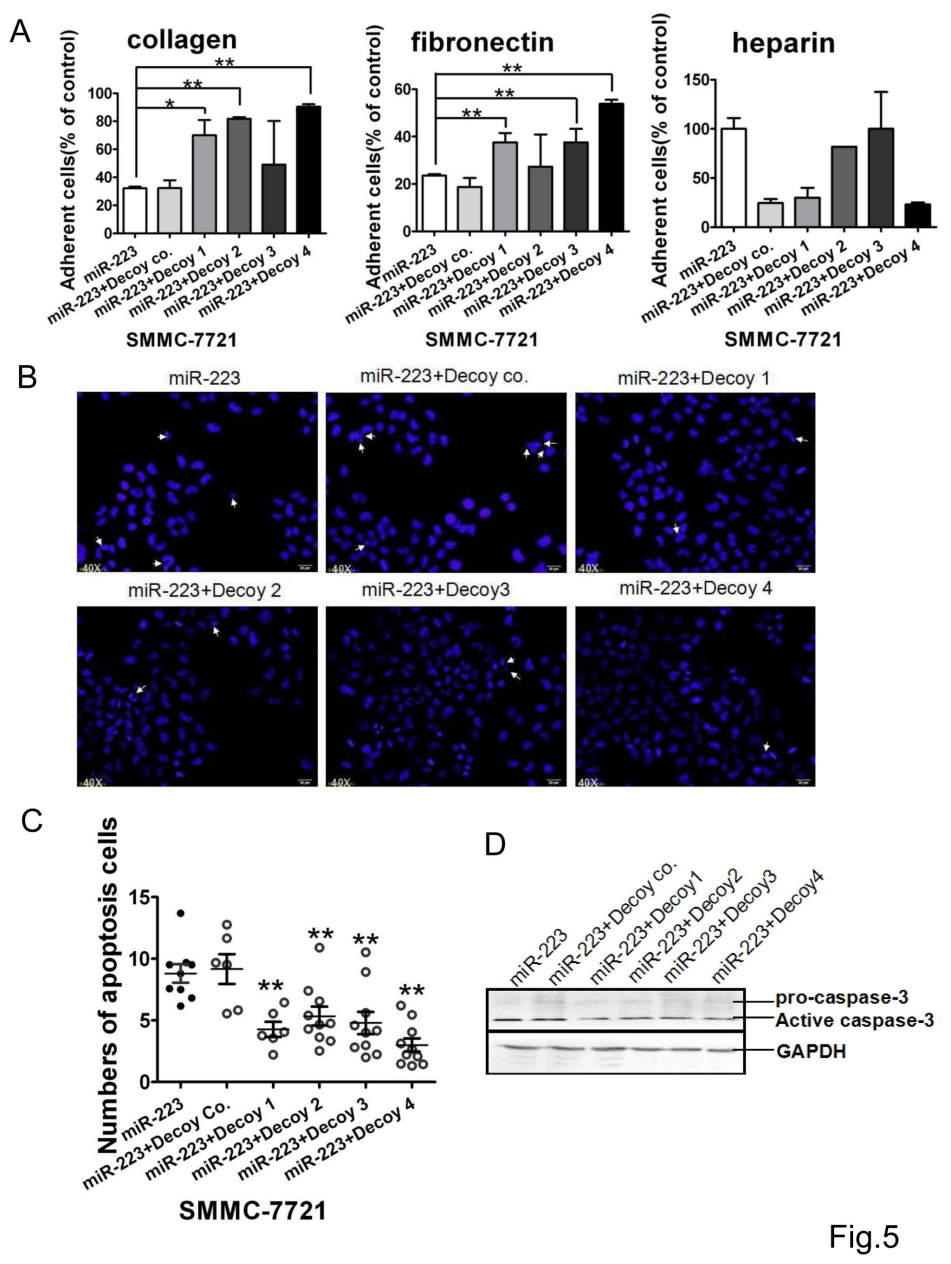
Decoy nucleotides reduced miR-223-mediated cell apoptosis. **A**. The adhesion to collagen type I (left), fibronectin (middle) and heparin (right) was assayed in SMMC-7721 cells co-transfected with miR-223 and decoy nucleotides. The adhesion percentage of the positive control was calculated from 3 independent experiments. **B**. Representative of micrograph images (40X) for the apoptosis cells stained with Hoechst33258 (white arrow) of SMMC-7721 co-transfected with miR-223 and decoys. **C**. The numbers of apoptosis cells were counted in 10 random view fields (left) under a microscope. In each field, 200 cells were counted and the number of apoptosis cells in every 200 cells was summarized and plotted. **D**. Caspase-3 analysis by Western in SMMC-7721 cells co-transfected with miR-223 and decoy nucleotides. GAPDH served as an interior loading control. Data are representative from 3 independent experiments. *, *P*<0.05, **, *P*<0.01, co, control.

Extracellular matrix collagen and fibronectin are important for cell adhesion. Hence, we further investigated the effect of these decoys on the cell adhesion to these proteins. The results of the cell adhesion assays showed that decoys 4 and 1 enhanced the adhesion ability of SMMC-7721 cells to collagen and fibronectin, which was inhibited by miR-223 (Figure 5A). Decoys 2 only enhanced the adhesion to collagen and decoy 3 to fibronectin. All the decoys had no significant effect on the cell adhesion to heparin (Figure 5A). The results of Hoechst staining showed that all the four decoys reduced the numbers of cell apoptosis induced by miR-223 (Figure 5B,C), but no significant difference among them although decoys 1 and 4 reduced the active caspase-3 more efficiently (Figure 5D). 

## Discussion

The miRNA can act like a siRNA and interfere with the expression of target mRNA[16]. One way to gain a better understanding of the role of a miRNA and demonstrate the network of miRNA-target complex is to disturb the miRNA. For example, antisense miRNA inhibitor is an effective approach that has been taken to verify the activity and function of the miRNA. Nevertheless, miRNAs do not encode amino acid sequence; they are not traditional therapeutic targets of small-molecule inhibitors [17]. Thus, to explore and seek molecule-based miRNA inhibitor presents significant challenges to researchers. The diverse targets of miRNA are reflected in the multi-biological regulation of cellular activities since each miRNA generally targets hundreds of mRNAs [18]. Current bioinformatics predictive algorithms show that there are many more potential targeting sites [19] than actual knowledge of a miRNA, and inhibition of one miRNA will affect many target molecules in consequence. To identify and screen the decoys that possess selective inhibitory effect on miRNA targeting is needed. Loss of miRNA function by complete complement antisense inhibitor causes expression changes of all the target molecules and leads to the reverse of all the actions of the miRNA in the regulation network. This may result in imbalance of the biological regulation and some of the regulation may not be our aim for the potential and clinic therapeutic application. It is needed to develop an approach of selective rescuing one mRNA from the targeting by a specific miRNA for the future application. Decreased inhibitor concentration was reported to be able to improve the specificity of the inhibitors [20], but this lead to diminish the inhibitory effects. Still, it remains unclear if there is a specific combination of length, sequences, structures, chemical modifications and location [21] that can significantly increase the potency of the inhibition. Current tools based on biochemistry for the sequence-specific inhibitors of miRNA are limited to single-stranded oligonucleotide-based miRNA inhibitors [22,23] which are complementary to the whole mature miRNA sequence, and probably affect irrelevant targets of the miRNA. Prior experimentation with antisense inhibitors demonstrated that they effectively inhibit miRISC particles in cytoplasmic extracts [23]. However, in general, miRNA inhibits its target primarily by seed sequence (from 2 to 7) matching to the 3’UTR of the target mRNA although the 3' region, from 13 to 18, of miRNA is also important for the specific and stable interaction between miRNA and 3’UTR [20]. 

In this study we designed 4 decoy oligonucleotides according to miRNA-binding sites of the target 3’UTR or the seed sequence of miR-223 with 2’-O-methylated modification. Using these synthetic decoys we tested their specificity and efficiency of the inhibitory effects on various mRNA 3’UTRs which were putatively targeted by miR-223, with luciferase reporter systems that are useful to confirm the predicted miRNA binding sites [24]. We also investigated the change of mRNA and protein by qPCR and western analysis to monitor miR-223 activity. Although the decoy 4, an antisense inhibitor complementary to the whole mature miR-223, showed the most potent suppression of the activity of miR-223, the suppression effect was seen in all targets of miR-223 tested, suggesting that the inhibition by full complementary oligonucleotides to mature miR-223 involved all the targeting activities of miR-223. The target decoy which is like competing RNA, has relatively distinctive and selective inhibition effect on the action of miR-223 according to complement sequence of different targets. Selmena et al. proposed that competing endogenous RNA (ceRNA) activity forms a large-scale regulatory network across the transcriptome [25]. Exogenous competing RNA, the decoys, will certainly involve the regulation by miRNA, but individually. Our data revealed that decoy 1 recovered the expression of IGF1R from miR-223 inhibition efficiently since it contained the sequence of IGF1R mRNA 3’UTR which was putatively bound by miR-223, while decoy 2 failed to rescue IGF1R expression efficiently. Decoy 1 was also able to restore FOXO3 and POLR3G expression from miR-223 inhibition since their binding sites of 3’UTR were similar with IGR1R. FOXO1 expression could not be rescued by decoy 1 as efficiently as by decoy 2, suggesting that the extra base pairs outside the seed sequence reduced the efficiency of decoy 1 rescue. Furthermore, decoy 1 did not rescue the expression of CDC27 and FBXW7 at all, indicating that decoy 1 was not able to rescue the expression of such mRNAs with more than 1 binding site for miR-223. These differences observed among different decoys inhibiting the same miRNA demonstrated that decoy 1 selectively attenuated miR-223 targeting IGF1R mRNA. Anti-miRNA nucleotides that were complementary to different regions of miRNA affected the decoy activities and resulted in differential inhibition efficiency. Pairing of only 6 - 7 bp in the seed region may be insufficient to form a stable miRNA-target complex and diminish the rescue efficiency, but can strengthen the rescue specificity. Additional base-paring around the seed sequence of miRNA might provide more affinity to ensure stable miRNA–RISC–decoy association and decoy 2 rescued FOXO1 expression more efficiently than decoy 1. Although the 5' end sequence of mature miRNA is functionally important, decoy 3 nucleotide binding 3’ flank region of miR-223 also showed potent rescuing effect, but the specificity reduced. The decoy binding to endogenous mature miRNA might be the same as miRNA to its target and is irreversible. Thus these decoys are presumed to sequester the endogenous miRNA, making it unavailable for their normal function [6,7]. To observe the cellular behavior alteration after the inhibition by miR-223, we tested these decoys in cell proliferation assays and found that the decoys 1 and 4 promoted cell growth significantly in LM3, SMMC-7721 and NB4 cells. 

In conclusion, our data demonstrated that sequence-specific oligonucleotide of IGF1R 3’UTR was able to recover IGF1R expression from miR-223 inhibition.
